# Identifying the Food Sources of Selected Minerals for the Adult European Population among Rice and Rice Products

**DOI:** 10.3390/foods10061251

**Published:** 2021-05-31

**Authors:** Joanna Bielecka, Renata Markiewicz-Żukowska, Patryk Nowakowski, Anna Puścion-Jakubik, Monika Grabia, Anita Mielech, Jolanta Soroczyńska, Katarzyna Socha

**Affiliations:** Department of Bromatology, Medical University of Białystok, Mickiewicza 2D Street, 15-222 Białystok, Poland; joanna.bielecka@umb.edu.pl (J.B.); patryk.nowakowski@umb.edu.pl (P.N.); anna.puscion-jakubik@umb.edu.pl (A.P.-J.); monika.grabia@umb.edu.pl (M.G.); anita.mielech@umb.edu.pl (A.M.); jolasor@interia.pl (J.S.); katarzyna.socha@umb.edu.pl (K.S.)

**Keywords:** trace elements, nutrients, rice, rice products, dietary sources, human health

## Abstract

The problem of dietary deficiency of several essential elements among different stages of life is still observed. The consumption of whole grains (among others unprocessed rice) is recommended as a part of a healthy diet. This research aimed to determine the content of selected macro- and microelements in rice and rice products to verify whether the tested products could be regarded as a source of selected minerals in the diet of the adult European population. Methods: A total of 99 samples from 12 groups of rice products (basmati, black, brown, parboiled, red, wild, white rice and expanded rice, rice flakes, flour, pasta, and waffles) were obtained. The atomic absorption spectrometry method (AAS) was used to determine the content of Ca, Cu, Fe, Mg, Mn, Se and Zn in the study material. Results: The average measured contents of Ca, Cu, Fe, Mg, Mn, Se and Zn were as follows: 226.3 ± 160.6 mg/kg, 3.6 ± 2.8 mg/kg, 9.4 ± 7.0 mg/kg, 618.0 ± 498.4 mg/kg, 16.7 ± 10.0 mg/kg, 242.9 ± 140.4 µg/kg and 19.5 ± 15.0 mg/kg, respectively. Statistical analyses confirmed the differences in the levels of the studied elements between the subgroups of processed and unprocessed products. Considering the tolerable upper intake level of studied elements, the tested products could be regarded as safe to consume. Conclusion: All tested products can be recommended as a source of Cu, Mn, and Se, while a majority of studied products can be considered a source of Mg and Zn in the diet of the adult European population.

## 1. Introduction

The consumption of whole grains (unprocessed maize, oats, wheat and unpolished rice) is recommended as a part of a healthy diet. According to the Food and Agriculture Organization (FAO, Geneva, Switzerland) statistics, in 2020, global cereal production reached 2.7 billion tonnes for which rice was the second-largest crop after wheat (764.9 and 509.1 mln of tonnes, respectively). The predominant part of the rice available on the world market is produced in Asian countries (>90%); only 0.6% is cultivated in European countries. The world’s three top producers of rice include China followed by India and Indonesia. The highest average rice consumption between 2016 and 2018 in Asian and Pacific countries was estimated at 77.6 kg per capita per year, while the lowest was in the European countries with 5.8 kg per capita per year [1].

Rice is one of the most important crops considering the total production as well as consumption worldwide. Two rice species—*Oryza sativa* S. (Asian rice) and *Oryza glaberrima* L. (African rice)—are cultivated. However, *O. sativa* is the predominant species, grown across the world; in turn, *O. glaberrima* is cultivated mainly in Africa and differs only slightly in its morphological aspects [2]. In the structure of paddy rice grains, two main parts can be specified: the hull and the caryopsis. The hull located outside the grain is rich in minerals and constitutes about 20% of the whole grain. The caryopsis is under the hull and includes the bran layer, endosperm and germ. The germ is the most nutritious part, rich in protein, fatty acids and mineral compounds [3]. Depending on the processes carried out on the grain, the rice can be classified as paddy (kernels are present inside the hull), brown (when the caryopsis is removed in the hulling process), white or milled (the milling process removes the bran layer and the germ from the brown rice) or parboiled (soaked in hot water then steamed before drying) [4]. White rice has a lower content of protein (especially a reduced lysine amount), minerals and fiber in comparison to brown rice. The parboiling process reduces the glycemic index and additionally contributes to the transfer of some minerals into the grain, which has a positive effect on its nutritional value [4].

It is observed that high consumption of white rice is related to increased risk of impaired glucose homeostasis and the occurrence of type 2 diabetes mellitus (T2DM). On the other hand, black rice intake is related to a lower postprandial blood glucose response (the glycemic index) and consequently, reduced risk of metabolic disorders, such as obesity, hyperglycemia, dyslipidemia, T2DM and hypertension. The anti-cancer activity of black rice bioactive compounds has also been demonstrated [4].

Well-balanced nutrition should provide not only adequate amounts of essential nutrients, such as proteins, carbohydrates and fats as well as vitamins and minerals. According to the available literature, the problem of unbalanced diets is still observed. The inadequate intake of macroelements such as calcium (Ca) or magnesium (Mg) was reported [5]. On the other hand, the over intake of copper (Cu) and manganese (Mn) were determined [6].

Ca plays a crucial role in the mineralization of bones and teeth, takes part in neuromuscular conduction and is required in maintaining the balance of body fluids within cells. Inadequate dietary intake of Ca may result in increased bone mass loss and osteoporotic fractures in old age [7].

Cu is essential for the functioning of several physiological pathways due to being a cofactor or structural component of many enzymes (e.g., cytochrome c oxidase, ceruloplasmin). Cu is important in the neuromodulation process, in response to low blood pressure as well as in angiogenesis. In the case of insufficient intake of Cu with diet, hematological manifestations (such as anemia), bone abnormalities or neurological symptoms may occur [8].

Iron (Fe) is one of the key elements in hematopoiesis. Furthermore, Fe is important for oxygen transport in the human body and is an important element in several metabolic processes, such as the synthesis of deoxyribonucleic acid (DNA) and the transport of electrons. Fe deficiency may lead to the development of anemia, which adversely influences cognitive functions, learning ability and immunity responses. Anemia is particularly dangerous in pregnancy because it is related to several negative outcomes, such as low birth weight or maternal and perinatal mortality [9].

Mg is involved in more than 300 biochemical reactions in the body as a cofactor of enzymes. It takes part, among others, in the production of energy, neuromuscular conduction, bone mineralization, DNA and RNA synthesis. Inadequate Mg intake increases the risk of the development of numerous chronic diseases, such as insulin resistance, T2DM, hypertension, Alzheimer’s disease or osteoporosis [10].

Mn is the next element, which participates in a variety of metabolic reactions in an organism. Mn is essential for the proper functioning of the immune, nervous and reproductive systems. Moreover, Mn plays a role in antioxidant responses, energy metabolism and inactivation of metalloenzymes and enzymes crucial for the synthesis of neurotransmitters. Insufficient Mn intake may be associated with impaired metabolism of proteins, fats and carbohydrates, growth disturbances and fertility problems. Excessive Mn intake may lead to the development of a neurodegenerative disorder, called manganism, which causes dopaminergic neuronal death [11].

Selenium (Se) is a trace element characterized by a narrow range between physiological status, deficiency and excessive (toxic) concentration. At the same time, Se is an important element in many physiological processes directly or indirectly. It constitutes a part of antioxidant enzymes (e.g., glutathione peroxidases), supports the response of the immune system and is required for maintaining reproductive health. Se is essential for proper thyroid functioning by being a part of iodotyrosine deiodinase, which catalyzes the deiodonization of thyroxine (T4) to triiodothyronine (T3). Considering the adverse health effects of inadequate Se intake, disorders of the heart muscle and joints are mainly observed. Increased risk of infertility and prostate cancer in men and neurological impairments are also possible [12].

Zinc (Zn) plays numerous roles in the human body, which could be classified as regulatory, catalytic and structural functions. Among the most important Zn functions, the effect on wound healing, supporting the immune and reproductive systems, regulation of blood pressure and heart rate as well as proper insulin secretion are described. Zn deficiency may impair the functioning of the immune, reproductive, nervous and gastrointestinal systems [13].

Because of the lack of favorable conditions to grow, rice species are not cultivated in Poland. Rice grains available on the Polish market are imported from different world regions, mainly from Asia, Southern Europe and Southern Africa. In this study, we were interested in the assessment of the nutritionally important components of which include macro- and microelements of different rice grains and rice products. To the best of our knowledge, such a broad group of different types of rice products has not yet been studied so far. We collected a broad range of diverse products, including seven rice subgroups and five subgroups of rice products.

In our research, we aimed to determine the content of selected macro- (Ca and Mg) and microelements (Cu, Fe, Mn, Se, and Zn) in rice and rice products, which has not been investigated yet. Moreover, we estimated whether the tested products can be regarded as a food source of the mentioned elements in the diet of the adult European population.

## 2. Materials and Methods

### 2.1. Sample Collection

The samples, representative of the overall Polish market, were obtained from local markets in north-eastern Poland. A total of 99 products were acquired between March and May 2020. We collected twelve subgroups of products (at minimum five samples each), among which seven were different types of rice: basmati (*n* = 10), black (*n* = 6), brown (*n* = 10), parboiled (*n* = 10), red (*n* = 5), wild (*n* = 5), white (*n* = 11). Moreover, we also obtained five different types of rice products: expanded rice (*n* = 8), rice flakes (*n* = 12), flour (*n* = 6), pasta (*n* = 7), and waffles (*n* = 9). The samples were not duplicated; each one of the products in the subgroups (e.g., among flakes) was purchased from a different producer. Considering the country of origin, the lack of this information in 19 products was observed. Most of the samples collected were imported from Asian countries (*n* = 53), while 27 were imported from Europe.

### 2.2. Sample Digestion

The preparation of samples for the mineralization process included homogenization in the stainless-steel mill. The samples were not treated thermally prior to the analysis. An appropriate amount (0.2–0.3 g) was weighted and transferred into mineralization vessels, then 4 mL spectrally pure concentrated (69%) nitric (V) acid was added (Tracepur, Merck, Darmstadt, Germany). A close-loop system was used to perform microwave digestion (Berghof, Speedwave, Eningen, Germany).

### 2.3. Analysis of Studied Elements Contents

The content of selected elements in the digested rice samples was determined by atomic absorption spectrometry (AAS), using the Z-2000 instrument (Hitachi, Tokyo, Japan). Before the analysis, the majority of the mineralized samples were diluted 20 times for Ca and Cu, 5 for Fe, 50 for Mg, 100 for Mn, 2 for Se and 10 for Zn. In the case of Cu, Mn, and Se, the flameless AAS technique with electrothermal atomization in a graphite cuvette was applied. To determine the Se content, the palladium–magnesium matrix modifier (Merck, Darmstadt, Germany) was added (Pd concentration: 1500 mg/L; Mg concentration: 900 mg/L), while for the Mn measurement, magnesium nitrate (Mg(NO_3_)_2_ concentration: 100 mg/L, Sigma-Aldrich, Merck, Darmstadt, Germany) as a modifier was used. The flame AAS technique in the acetylene–air flame with Zeeman background correction for the determination of Ca, Fe, Mg, Zn was used. Moreover, in the case of Ca and Mg, 1% lanthanum chloride (LaCl_3_, Sigma-Aldrich, Merck, Darmstadt, Germany) was adopted as a masking agent. The analytical conditions of the process of the flameless and flame techniques are presented in Table 1.

### 2.4. Method Validation

The certified reference materials (CRMs) were used to control the quality of performed analyses (Table 2). In the case of Ca, Cu, Fe, Mg, Mn, and Zn, corn flour (INCT–CF-3) was applied, while for Se, mushroom powder (CS–M-3) was used (both standards produced by the Institute of Nuclear Chemistry and Technology, Warsaw, Poland). The CRMs were analyzed before and for every ten determinations.

### 2.5. Assessment Whether Tested Products Could Be Regarded as a Source of Studied Nutrients

We assessed whether the tested groups of products contained in 100 g, at minimum, 15% of the reference value intake (RVI) established by the European Parliament and of the Council. If this requirement was met, the product could be regarded as a source of the studied elements for the adult European population [14].

### 2.6. Estimation of the Risk of Adverse Health Effects

Based on the results obtained in this research and the tolerable upper intake level (TUIL) determined by the National Institutes of Health in the United States of America, the risk of adverse health effects resulting from daily consumption of the studied nutrients was evaluated. The limits considered the highest daily nutrient intake, which should pose no adverse health effects to almost all individuals in the general population. The following levels for adults over 19 years of age were established: Cu, 10 mg/day; Fe, 45 mg/day; Mn, 11 mg/day; Se, 400 µg/day; and Zn, 40 mg/day. In the case of Ca, two age groups were specified: 2500 mg/day (for adults aged 19–50 years) and 2000 mg/day (for adults over 50 years). For Mg, the TUIL was given for pharmacological agents use and did not include intake from food and water; therefore, it was not analyzed in our research [15]. The maximum amounts of safe intake levels of studied products were calculated.

### 2.7. Statistical Analyses

Statistica software (Tibco, Palo-Alto, CA, U.S.A.) was used to analyze the data. The normality distribution of the data was checked by the Shapiro–Wilk test. The results were shown as mean (X) with standard deviation (SD), minimum (Min), maximum (Max) as well as the median and interquartile range (lower quartile Q_1_, upper quartile Q_3_). Kruskall–Wallis Analysis of Variance (ANOVA) with post-hoc analysis to compare the content of the studied elements between the subgroups of products was performed. Significant differences were assumed at *p*-values < 0.001, < 0.01 and < 0.05.

## 3. Results

The results obtained in our analysis are presented in Table 3 and Table 4.

None of the subgroups tested can be considered a source of Ca and Fe in the diet of the adult European population (the RVI was lower than 15%). Contrastingly, every studied subgroup could be taken into account as a source of Cu, Mn, and Se. In the case of Mg, six subgroups (basmati, expanded, parboiled, white rice, flakes and pasta) and three of Zn (basmati, parboiled rice and pasta) did not meet the requirements; therefore, they cannot be regarded as a source of these nutrients in the diet (Table 3 and Table 4).

The TUIL for Ca in every subgroup was higher than 7000 g/day, while that for Fe was higher than 2570 g/day. TUIL for Cu ranged from 811 g/day for wild rice to 4579 g/day for white rice; for Mn, it was lowest for red rice (298 g/day) and highest for parboiled rice (5300 g/day). In the case of Se, the lowest TUIL was calculated for parboiled rice (1297 g/day) and at the same time, the highest (2321 g/day) for wild rice. The TUIL for Zn ranged from 672 g/day for wild rice to 7265 g/day for parboiled rice.

The statistical analysis confirmed the differences in the content of studied elements between the unprocessed (e.g., black or red rice) and processed (e.g., flakes or pasta) subgroups of products. The Kruskall–Wallis Analysis of Variance (ANOVA) test with post-hoc analysis was used. Only in the case of Cu, no differences were found between the studied products. The differences between the contents of the studied elements in the studied product groups are shown in Table 5. The *p*-values (*p* < 0.05, 0.01, 0.001) were placed in superscript.

## 4. Discussion

In this investigation, we assessed the quality of 12 different groups of rice products available on the Polish market by determining the content of macro- and microelements. The results obtained by other authors are presented in Table 6.

Compared to other plant products considered to be sources of Ca (such as legumes), the Ca content in our study material was generally low; however, most of the other authors [16,19,20,23,25] observed even lower Ca levels than that measured in our samples ( Table 3 and Table 6). Only Pinto et al. determined in parboiled rice a higher Ca level than that in this research [18]. The parboiling process increases the Ca content due to specific conditions, such as soaking time and proper temperature [27]. The tested products could not be regarded as a source of Ca in the diet of European adults (Table 3). Insufficient Ca intake is still a worldwide problem. Taking into account Ca intake with a whole day diet, most of the available research determines the insufficient intake of this nutrient [5,28]. In the study by Grygorieva et al., among Ukrainian adults, only 7.8% of subjects consumed more than 1200 mg of Ca per day [29]. The problem of inadequate Ca consumption amid Spanish adolescents and elderly people population was observed as well [30]. Considering the daily intake levels, Kashian and Fathivand observed that rice consumption fulfilled the recommended dietary allowances (RDA) norm within 1% of the Iranian population [23]. Similar observations were found in the study conducted by Pinto et al. among the Iberian (Portugal and Spain) population; daily Ca requirements by rice consumption were met from 0.2% to 1.3% of the RDA norm [18]. Other Spanish researchers also reported that daily Ca intake through rice consumption ranged from 0.6% of RDA norm for white rice to 1.07% of RDA for brown rice. However, when brown rice was replaced by white rice, the daily Ca intake was reduced by 47% [16].

The Cu levels measured in this investigation are in agreement with those reported by other authors [16,17,18,19,20,21,22,24,25,26]. Taking into account the recommendations of Cu intake, rice products available on the Polish market could be regarded as a source of this element in the daily diet of European adults (Table 3). Regarding rice consumption, observations were made by Antoine et al. that among the Jamaican population, polished rice intake fulfilled daily Cu requirements in 13%, while unprocessed grains in 23% [19]. Among Malawian adults, daily rice intake covered 19% of the RDA, while amid the Nigerian population, 31% [20,25]. Lower daily Cu intake was reported for Iberian populations and ranged from 6.4% to 13% of the RDA norm [18]. Jo and Todorov demonstrated that in rice grains, Cu is concentrated mainly in the bran and outer layers of the endosperm. When brown rice was polished to white rice (removal 18% of grain weight), the Cu content decreased by nearly 20% from 2.29 mg/kg to 1.91 mg/kg [31]. Moreover, Hensawang et al. reported that extra thorough rice polishing reduced Cu content by 35% [32]. Ortiz and Camara-Martos determined Cu bioaccessibility through solubility and dialyzability assays among eight of the most consumed rice varieties in Argentina and Spain. The bioaccessibility ranged from 24% to 80%, while dialyzability ranged from 4% to 41% [33]. In the study by Babaali et al., among the Iranian population, the average daily Cu intake exceeded the estimated average requirements (EAR) and was determined at 3.8 mg; fresh fruit and vegetables as the main sources (31%) of this element were indicated [34]. More than three times lower dietary Cu consumption (1.2 mg/day) was reported in the investigation conducted among Japanese adults. The authors observed that white rice accounted for approximately 20–30% of the total intake of this element [35].

Considering the Fe content in rice samples, some other authors [16,17,18] reported comparable results to ours (Table 3 and Table 6). However, higher Fe levels were also frequently reported [19,20,21,23,24]. None of the subgroups tested can be a source of Fe for the adult European population (Table 3). Women of childbearing age are at risk of inadequate Fe intake due to high body requirements for this element. Considering Fe intake with whole day diet among Spanish adults, only 17% of women had sufficient intake, while among men, it was 57.3% of participants [36]. Similar observations were reported among Polish adolescents; females had significantly lower Fe intake than males [37]. On the other hand, both white and brown rice fulfilled daily RDA for men in Jamaica at 20% and 18%, while for women it was 8.8% and 8%, respectively [19]. Among the Iranian population, rice consumption covered the recommended daily intake in 14% for women and 31% for men [23]. Fe in rice grains is unevenly distributed and located mainly in the pericarp and aleurone layers. Five times higher Fe concentration was observed in the dorsal compared to the ventral side of the bran layer. The polishing process (18% removal of grain weight) reduced Fe content in brown rice from 10.4 mg/kg to 3.6 mg/kg in white rice; therefore 66% of Fe was removed [31]. Besides the total content of Fe in rice, the presence of compounds negatively affecting its bioavailability (such as phytic acid, polyphenols, tannins and dietary fiber) should be taken into account. Average Fe solubility was within the range of 80% in rice grains, except for brown rice. The lowest bioaccessible percentage, despite the high Fe content, was a result of a high concentration of the antinutritional factors mentioned above [33].

The Mg levels determined in our research (Table 3) were close to those reported in other investigations [16,18,20,24]. Except for six groups of products (basmati, expanded, parboiled, white rice, flakes and pasta), the remaining rice products can be a source of Mg for European adults (Table 3). Taking into account the daily rice consumption, Portuguese and Spanish citizens fulfilled the RDA norm from 2.4% to 11.8% among women and between 1.8% and 9% among men. Amid the Jamaican population, white rice consumption covered Mg daily requirements in 8.2% in females and 6.3% in males, while brown rice in 27% and 20%, respectively [19]. Cano-Lamarid et al. observed that the replacement of brown rice with white rice in the diet may result in a reduction in daily Mg intake by 70% [16]. Daily Mg intake was generally adequate in research conducted by Schiefermeier-Mach et al.; however, in nearly one-third of participants, the coverage of requirements was not met [38]. Similar results were observed among the Iranian population—22.4% of the study group had an Mg intake lower than the EAR norm [34].

A lower Mn content than found in this investigation was reported by Pinto et al. in the case of white and wild rice, and by Halder et al. also, in white rice [18,24], whereas most of the other researchers determined similar levels of Mn in rice samples [16,17,19,20,21,22,25,26]. Every subgroup of the rice products tested can be a rich source of Mn in the diet of the adult European population (Table 4). One portion (300 g) of red rice reached the TUIL of Mn in our investigation. Moreover, when another foodstuff consumed daily is added, there is a potential risk of elevated Mn levels in the body, which could pose negative effects. The investigation by Choi and Bae among Korean adults demonstrated that Mn intake with diet covered the adequate intake (AI) norm in 103% for men and 110% for women and the main sources of this element were cereal and cereal products [39]. Regarding the daily intake, in Antoine’s et al. investigation, white rice consumption fulfilled the recommended level of intake for women at 41% and men at 32%, while brown rice at 105% and 82%, respectively [19]. Pinto et al. estimated that the AI norm for the Iberian females through rice consumption was covered from 10.8% to 42.5% and from 8.5% to 33.3% for Iberian males [18]. The total content of Mn in the investigation conducted by Ortiz and Camara-Martoz ranged from 2.5 mg/kg to 14 mg/kg. Taking into account the bioaccessibility, the concentration of soluble Mn varied from 0.35 mg/kg to 2.52 mg/kg, while the dialyzable fluctuated from 0.15 mg/kg to 0.64 mg/kg. Moreover, the authors observed a statistically significant negative interaction between the content of soluble Mn and fiber in the studied grains [33]. Hensawang et al. found that the polishing process decreased the Mn concentration by 62% [32]. The content in unpolished and polished grains differed significantly (*p* < 0.01, r = −0.747). On the other hand, Jo and Todorov reported lower Mn reduction (43%) during the polishing process. The Mn in rice grain, similar to Fe, is localized mainly in the pericarp and aleurone layers; however, this trace element is also present in the endosperm. In the dorsal, compared to the ventral, side of the bran layer, a five times higher Mn concentration was observed [31].

Considering Se levels in rice products, our results were higher than reported by most of the other researchers [17,18,19,20,22,26]. Only Halder et al. measured a fairly similar content, while Aderide et al. determined greater amounts of Se in tested rice [24,25]. Due to considerably high Se levels, every subgroup of the rice products tested can be regarded as a source of this trace element for European adults (Table 4). Cereals and meat were indicated as the main sources of Se in the diet of Italian adults. The daily level of Se consumption was sufficient; for women, it was estimated at 65 µg/day and for men at 67 µg/day [40]. Similar results were observed among Belgian adults [41]. Taking into account rice consumption, among the Brazilian population, rice intake covered 5% of the daily Se requirement [25]. For Jamaican adults, brown rice consumption covered 17% of the daily norm, while white rice covered 14% [19]. In the research conducted by Jo and Todorov, the polishing process slightly decreased Se content in whole grain rice from 150 µg/kg to 144 µg/kg in white rice. Additional analysis showed that Se is evenly distributed through the grain [31].

The Zn content in this study (Table 4) was comparable to the results obtained by most of the other authors cited in Table 6 [17,18,19,20,21,23]. In three studies, the measured Zn levels were lower than observed in our samples [24,25,26]. Only Rothenberg et al. reported higher Zn levels [22]. Except for basmati and parboiled rice as well as pasta, the remaining studied products could be a source of Zn for the adult European population (Table 4). In the investigation of Pinto et. al, consumption of rice fulfilled the RDA norm by 1.8% to 10.9% [18]. Fairly similar fulfilment of the RDA norm (9%) was observed amid Nigerian women [25]. During the polishing process, the content of Zn was reduced by 23% (from 18.3 mg/kg to 14.1 mg/kg). Generally, Zn is distributed homogeneously through the grain; however, the bran layer contains higher average Zn levels (20–80 mg/kg) compared to the endosperm (13 mg/kg) [31]. The high content of antinutritional components in bran impaired Zn bioaccessibility from rice grains. In the study by Ortiz and Camara-Martoz, the total Zn level ranged from 8.8 mg/kg to 13 mg/kg and the following soluble and dialyzable contents were reported: 0.9–3.3 mg/kg and 0.2–1.3 mg/kg, respectively. Thus, Zn bioaccessibility was quite low—the solubility did not exceed 15%—while dialyzability was lower than 3%. It was observed that vegetable proteins were negatively correlated (*p* < 0.05, r = −0.409) with Zn bioaccessibility [33]. Considering the overall daily diet, among Chinese adults, over one-third are at risk of Zn deficiency due to intake below the EAR norm. Grain consumption contributed to nearly 40% of total dietary Zn intake [42]. On the other hand, among American adults, daily Zn intake above RDA norm was reported [43].

To the best of our knowledge, there are limited data regarding the TUIL for elements consumed with diet. The risk of Ca intake with food products reaching TUIL is very low [44]. In a large European cohort study, exceedances of TUIL considering Fe (among women and men) and Mg (among men) were reported. The risk of over intake was observed among those who consumed dietary supplements [45]. No nutrient intakes with a diet above the TUIL were reported among the Canadian population. However, study participants who took dietary supplements were at risk of overly high Mg and Zn intake [46]. In turn, our analyses showed that there could be a potential risk of over intake of Mn through the whole day diet (even without supplementation). Therefore, we see a great need to conduct research focused on this topic.

## 5. Conclusions

Taking all of the above described into account, our study revealed that all of the studied subgroups of rice products available on the Polish market can be regarded as a source of Cu, Mn, and Se, while most of the products can be a source of Mg and Zn in the diet of the adult European population. Therefore, this fact should be highlighted in nutritional education. Considering the tolerable upper intake level of the studied elements, the tested products could be regarded as safe to consume. However, we see a great need to assess the daily intake of Mn with all products consumed in the diet due to the possible risk of excessive intake.

## Figures and Tables

**Table 1:** The analytical conditions of flameless AAS technique (Cu, Mn and Se) and flame AAS technique (Ca, Fe, Mg and Zn) in determining the content of elements in rice samples.

Element							
Parameter	Cu	Mn	Se	Ca	Fe	Mg	Zn
Wavelength (nm)	324.8	279.5	196.0	422.7	248.3	285.2	213.9
Lamp current (mA)	7.5	7.5	14.5	7.5	12.5	7.5	6.5
Drying (°C)	80/140	80/140	70/100	-	-	-	-
Ashing (°C)	600/600	750/750	600/600	-	-	-	-
Atomization (°C)	2400/2400	2300/2300	2700/2700	-	-	-	-
Cuvette cleaning (°C)	2500/2500	2500/2500	2800/2800	-	-	-	-

**Table 2:** The results obtained in the method validation.

Element	Detection Limit for Method *	Detection Limit for Samples ^1^	Recovery for CRM	Precision (%)
**Ca**	0.10 mg/L	9.26 mg/kg	98.4	3.6
**Cu**	0.65 µg/L	0.24 mg/kg	99.2	2.1
**Fe**	0.11 mg/L	1.29 mg/kg	98.7	2.5
**Mg**	0.009 mg/L	3.33 mg/kg	98.8	2.7
**Mn**	0.14 µg/L	0.13 mg/kg	101.2	2.4
**Se**	1.55 µg/L	57 µg/kg	97.5	4.7
**Zn**	0.015 mg/L	1.39 mg/kg	100.9	1.8

* Estimated as a characteristic concentration in standard solution for 0.0044 absorbance. ^1^ Detection limit considering amount weighted and diluted for samples.

**Table 3:** The content of studied elements (Ca, Cu, Fe, Mg) measured in rice and rice products.

The Type of the Rice and Rice Product	*n*	Ca (mg/kg)			Cu (mg/kg)			Fe (mg/kg)			Mg (mg/kg)		
		X ± SD (Min–Max)	Me (Q_1_–Q_3_)	% of RVI (800 mg)	X ± SD (Min–Max)	Me (Q_1_–Q_3_)	% of RVI (1 mg)	X ± SD (Min–Max)	Me (Q_1–_Q_3_)	% of RVI (14 mg)	X ± SD (Min–Max)	Me (Q_1_–Q_3_)	% of RVI (375 mg)
Basmati	10	193.5 ± 61.9 (133.3–343.9)	181.9 (151.8–208.3)	2	2.4 ± 0.4 (1.9–3.1)	2.2 (2.0–2.6)	24	6.5 ± 7.9 (2.4–28.9)	4.0 (3.4–5.1)	5	379.0 ± 378.6 (143.0–1363.0)	205.0 (176.0–532.4)	10
Black	6	324.1 ± 156.1 (73.4–443.5)	405.9 (185.5–430.7)	4	3.4 ± 1.5 (2.4–6.4)	2.9 (2.6–3.4)	34	16.7 ± 3.2 (12.1–21.7)	16.9 (15.0–17.7)	12	1167.8 ± 280.5 (997.1–1723.9)	1034.3 (1030.4–1186.9)	31
Brown	10	325.8 ± 162.2 (65.1–613.8)	329.2 (268.8–408.6)	4	4.0 ± 3.6 (1.3–13.1)	2.4 (1.7–5.0)	40	15.9 ± 3.1 (12.7–23.1)	15.1 (14.0–15.8)	11	1017.0 ± 386.5 (149.3–1468.5)	964.0 (882.0–1351.5)	27
Parboiled	10	137.5 ± 127.2 (23.7–463.7)	89.4 (68.8–175.0)	2	2.4 ± 0.5 (1.4–3.3)	2.5 (2.1–2.7)	24	6.5 ± 6.3 (1.8–22.8)	3.9 (3.1–6.9)	5	224.9 ± 87.1 (7.1–312.4)	239.5 (211.9–283.6)	6
Red	5	216.7 ± 227.6 (51.3–545.1)	66.0 (53.7–367.2)	2	3.7 ± 1.3 (2.3–5.5)	3.4 (2.8–4.3)	37	11.8 ± 3.2 (8.7–15.5)	10.0 (9.9–15.1)	9	1241.7 ± 305.6 (1014.6–1754.3)	1086.0 (1061.5–1292.3)	33
Wild	5	294.6 ± 143.5 (83.7–421.2)	367.2 (209.6–391.5)	5	12.6 ± 2.4 (10.5–16.6)	11.7 (11.5–12.8)	126	17.9 ± 3.1 (14.0–21.8)	17.7 (16.1–20.0)	13	1043.6 ± 155.1 (873.7–1292.3)	1014.0 (977.7–1060.0)	28
White	11	114.1 ± 72.6 (16.1–278.2)	88.2 (66.9–150.1)	1	2.4 ± 0.7 (1.4–3.8)	2.4 (1.7–2.9)	24	4.5 ± 2.9 (1.4–9.9)	3.7 (2.0–5.2)	3	258.2 ± 101.4 (109.6–423.8)	234.0 (181.7–371.9)	7
Expanded	8	187.0 ± 118.0 (74.0–406.2)	170.1 (81.5–256.3)	2	2.9 ± 1.9 (1.7–7.6)	2.4 (1.8–2.7)	29	4.8 ± 2.7 (2.1–10.6)	4.4 (2.9–5.6)	3	412.0 ± 138.3 (262.2–703.4)	415.3 (305.6–444.5)	11
Flakes	12	156.3 ± 94.01 (63.1–362.6)	129.5 (107.2–158.0)	2.	3.3 ± 1.2 (2.1–6.5)	3.0 (2.6–3.7)	33	5.5 ± 5.9 (1.4–18.2)	3.3 (3.0–4.2)	4	319.8 ± 318.0 (91.7–1086.2)	200.1 (137.4–305.7)	9
Flour	6	192.1 ± 132.3 (65.7–426.3)	185.8 (67.7–221.3)	2	2.4 ± 0.5 (1.7–3.0)	2.4 (2.2–2.7)	24	8.7 ± 5.1 (4.5–18.1)	7.4 (4.7–10.3)	6	571.4 ± 416.1 (264.8–1385.1)	433.6 (333.4–578.2)	15
Pasta	7	276.8 ± 279.5 (73.5–867.0)	185.6 (85.6–355.2)	3	2.4 ± 0.7 (1.4–3.2)	2.8 (1.7–2.9)	24	3.9 ± 1.5 (2.1–6.3)	3.5 (2.4–5.6)	3	125.7 ± 59.5 (81.6–250.4)	107.7 (86.9–146.7)	3
Waffles	9	402.7 ± 118.2 (134.3–574.7)	421.6 (379.3–437.7)	5	4.9 ± 3.1 (1.6–12.3)	4.2 (3.4–5.1)	49	17.8 ± 5.1 (12.5–28.8)	17.9 (14.0–20.1)	13	1361.9 ± 283.6 (929.4–1811.5)	1383.0 (1187.4–1544.1)	36
TOTAL	99	226.3 ± 160.6 (16.1–867.0)	176.1 (85.6–362.6)	-	3.6 ± 2.8 (1.3–16.6)	2.7 (2.1–3.4)	-	9.4 ± 7.0 (1.4–28.8)	5.9 (3.4–15.1)	-	618.0 ± 498.4 (7.1–1811.5)	382.3 (211.9–1030.4)	-

X—mean, SD—standard deviation, Min—minimum, Max—maximum, Me—median, Q_1_—lower quartile, Q_3_—upper quartile, RVI—reference value intake, calculated per 100 g of product.

**Table 4:** The content of Mn, Se and Zn in rice and rice products.

The Type of the Rice and Rice Product	*n*	Mn (mg/kg)			Se (µg/kg)			Zn (mg/kg)		
		X ± SD (Min–Max)	Me (Q_1_–Q_3_)	% of RVI (2 mg)	X ± SD (Min–Max)	Me (Q_1_–Q_3_)	% of RVI (55 µg/kg)	X ± SD (Min–Max)	Me (Q_1_–Q_3_)	% of RVI (10 mg)
Basmati	10	10.3 ± 4.2 (0.9–16.0)	10.3 (8.7–13.3)	52	303.1 ± 129.0 (213.3–630.7)	248.1 (222.1–313.8)	55	14.5 ± 1.7 (12.4–17.8)	14.5 (13.3–15.5)	14
Black	6	27.9 ± 5.4 (22.1–37.7)	26.9 (24.9–28.9)	139	185.2 ± 25.1 (157.6–229.0)	176.7 (173.2–197.7)	34	26.0 ± 3.3 (21.5–29.8)	26.6 (22.8–28.7)	26
Brown	10	24.6 ± 7.2 (11.5–35.7)	25.1 (22.0–29.3)	123	205.5 ± 98.8 (152.1–472.0)	168.1 (164.0–172.4)	37	26.0 ± 26.5 (10.4–100.3)	18.6 (15.4–22.1)	26
Parboiled	10	6.4 ± 4.8 (0.3–15.8)	4.7 (4.2–7.0)	32	391.8 ± 190.4 (167.6–673.2)	371.5 (219.3–569.0)	71	6.7 ± 2.9 (2.4–12.9)	6.0 (5.2–6.8)	7
Red	5	38.2 ± 8.7 (31.4–53.0)	36.2 (32.7–37.5)	191	194.5 ± 9.8 (180.4–204.3)	195.6 (189.5–202.6)	35	28.1 ± 7.7 (22.0–39.4)	23.8 (22.4–33.1)	28
Wild	5	16.1 ± 2.3 (13.9–19.8)	16.0 (14.4–16.4)	81	173.0 ± 11.1 (153.9–183.1)	175.9 (175.2–176.9)	32	63.3 ± 17.2 (44.6–85.4)	62.7 (48.7–74.9)	63
White	11	13.6 ± 4.6 (8.2–22.2)	13.1 (9.6–16.3)	68	214.2 ± 34.6 (170.7–275.0)	203.6 (188.9–244.5)	39	16.3 ± 2.0 (13.7–20.3)	15.9 (14.8–17.3)	16
Expanded	8	13.8 ± 4.1 (8.8–20.1)	14.0 (9.8–16.7)	69	247.7 ± 92.2 (164.5–463.5)	229.3 (196.3–251.1)	45	16.4 ± 4.2 (10.7–22.4)	15.0 (13.6–20.7)	16
Flakes	12	12.7 ± 5.4 (7.8–26.5)	11.1 (8.8–14.8)	64	196.9 ± 26.7 (171.8–244.5)	186.8 (179.0–212.2)	36	15.8 ± 4.0 (9.4–22.6)	15.01 (13.5–18.3)	16
Flour	6	15.7 ± 9.4 (8.2–34.1)	12.1 (11.4–16.1)	78	381.2 ± 389.0 (188.7–1174.1)	236.1 (206.2–246.1)	69	15.2 ± 2.4 (12.4–19.0)	15.2 (12.9–16.6)	15
Pasta	7	7.7 ± 3.8 (4.0–15.8)	7.4 (5.8–7.6)	39	211.4 ± 26.2 (163.5–234.8)	219.7 (188.3–232.4)	38	11.3 ± 5.3 (5.5–19.7)	10.2 (6.5–16.5)	11
Waffles	9	27.4 ± 6.1 (23.8–42.7)	25.0 (24.0–27.8)	137	181.1 ± 22.2 (150.1–207.2)	187.6 (155.7–197.3)	33	19.8 ± 4.0 (12.6–26.3)	20.2 (17.3–22.0)	20
TOTAL	99	16.7 ± 10.0 (0.3–53.0)	14.1 (8.8–24.0)	-	242.9 ± 140.4 (150.1–1174.1)	200.5 (176.9–241.5)	-	19.5 ± 15.0 (2.4–100.3)	16.1 (12.9–21.7)	-

X—mean, SD—standard deviation, Min—minimum, Max—maximum, Me—median, Q_1_—lower quartile, Q_3_—upper quartile, RVI—reference value intake, calculated per 100 g of product.

**Table 5:** The statistically relevant differences in the content of the studied elements considering the type of product.

	Basmati	Black	Brown	Parboiled	Wild	Flakes	Pasta	Waffles
Black				Mn^0.001^				
				Zn^0.001^				
Brown	Se^0.01^			Mn^0.001^		Fe^0.01^	Fe^0.05^	
				Se^0.05^			Mg^0.01^	
				Zn^0.01^			Mn^0.01^	
Red	Mn^0.01^			Mn^0.001^		Mg^0.05^	Mg^0.001^	
				Zn^0.05^		Mn^0.05^	Mn^0.001^	
							Zn^0.001^	
Wild	Se^0.05^			Zn^0.001^		Fe^0.05^	Mg^0.01^	
	Zn^0.05^						Zn^0.01^	
White		Fe^0.05^	Fe^0.05^	Se^0.05^	Fe^0.05^			Ca^0.01^
								Fe^0.01^
								Mg^0.01^
Flakes		Mg^0.05^						Ca^0.05^
								Fe^0.01^
								Mg^0.01^
Pasta		Mg^0.001^						Fe^0.05^
		Mn^0.01^						Mg^0.001^
		Zn^0.001^						Mn^0.001^
Waffles	Mg^0.001^			Ca^0.05^				
	Se^0.05^			Mg^0.001^				
				Se^0.05^				
				Zn^0.001^				

**Table 6:** Content of elements in rice and rice products determined by other authors.

Type of Product [Ref.]	*n*	Element						
		Ca (mg/kg)	Cu (mg/kg)	Fe (mg/kg)	Mg (mg/kg)	Mn (mg/kg)	Se (µg/kg)	Zn (mg/kg)
**Brown Rice**								
[16]	9	Min–Max: 87.1–114	Min–Max: 1.3–4.6	Min–Max: 13.3–14.5	Min–Max: 160–1630	Min–Max: 7.6–11.8	-	-
[17]	6	-	X = 3.1	X = 11.5	-	X = 28.9	X = 104	X = 22.5
[18]	11	X = 64±9	X = 1.6 ± 0.4	X = 14.0 ± 2.1	X = 1064 ± 87	X = 21.5 ± 4.4	X = 30 ± 20	X = 15.9 ± 2.3
[19]	16	X = 104 ± 37.9	X = 3.0 ± 1.1	X = 20.1 ± 7.8	X = 1205 ± 335	X = 26.5 ± 12.2	X = 131 ± 57	X = 20.2 ± 2.73
[20]	33	X = 72.6 ± 32.6	X = 5.5 ± 5.4	X = 32 ± 26.1	X = 1140 ± 214	X = 20.1 ± 10.5	X = 41 ± 57	X = 18.8 ± 4.3
		(35.6–211.5)	(Min–Max: 2.2–29.1)	(Min–Max: 12.1–119.1)	(Min–Max: 761–1550)	(Min–Max: 12.4–74.4)	(Min–Max: 5–300)	(Min–Max: 13.9–34)
[21]	51	-	X = 2.35	X = 18.6	-	X = 15.5	-	X = 21.0
			(Min–Max: 1.4–3.9)	(Min–Max: 10.0–65.2)		(Min–Max: 8.2–24.2)		(Min–Max: 9.0–29.4)
[22]	51	-	X = 4.4	-	-	X = 20	X = 39	X = 28
			(Min–Max: 0.9–6.5)			(Min–Max: 10–34)	(Min–Max: 15–80)	(Min–Max: 20–36)
Our results	10	X = 325.8 ± 162.2	X = 4.0 ± 3.6	X = 15.9 ± 3.1	X = 1017.0 ± 386.5	X = 24.6 ± 7.2	X = 205.5 ± 98.8	X = 26.0 ± 26.5
		(Min–Max: 65.1–613.8)	(Min–Max: 1.3–13.1)	(Min–Max: 12.7–23.1)	(Min–Max: 149.3–1468.5)	(Min–Max: 11.5–35.7)	(Min–Max: 152.1–472.0)	(Min–Max: 10.4–100.3)
**White Rice**								
[17]	5	-	X = 2.3	X = 3.7	-	X = 7.8	X = 92	X = 13.1
[18]	56	X = 32 ± 18	X = 1.8 ± 0.6	X = 6.8 ± 1.5	X = 225 ± 63	X = 1.8 ± 0.6	X = 200 ± 190	X = 13.5 ± 3.4
[19]	9	X = 127 ± 141 X = 37.7 ± 9.1	X = 1.7 ± 0.6	X = 22.3 ± 37.9	X = 371 ± 127	X = 10.5 ± 3.7	X = 108 ± 66	X = 15.6 ± 1.9
[20]	21	(Min–Max: 18.6–63)	X = 3.1 ± 2.0	X = 7.9 ± 2.9	X = 259 ± 44	X = 9.4 ± 1.7	X = 40 ± 35	X = 14.7 ± 1.8
		X = 114.1 ± 72.6	(Min–Max: 0.9–8.1)	(Min–Max: 3.6–17.3)	(Min–Max: 191–341)	(Min–Max: 7.0–12.7)	(Min–Max: 13–137)	(Min–Max: 12–17.7)
Our results	11	(Min–Max: 16.1–278.2)	X = 2.4 ± 0.7	X = 4.5 ± 2.9	X = 258.2 ± 101.4	X = 13.6 ± 4.6	X = 214.2 ± 34.6	X = 16.3 ± 2.0
			(Min–Max: 1.4–3.8)	(Min–Max: 1.4–9.9)	(Min–Max: 109.6–423.8)	(Min–Max: 8.2–22.2)	(Min–Max: 170.7–275.0)	(Min–Max: 13.7–20.3)
**Parboiled Rice**								
[18]	13	X = 370 ± 272	X = 2.0 ± 0.6	X = 8.6 ± 1.9	X = 370 ± 272	X = 5.6 ± 1.3	X = 100 ± 50	X = 6.1 ± 1.4
Our results	10	X = 137.5 ± 127.2	X = 2.4 ± 0.5	X = 6.5 ± 6.3	X = 224.9 ± 87.1	X = 6.4 ± 4.8	X = 391.8 ± 190.4	X = 6.7 ± 2.9
		(Min–Max: 23.7–463.7)	(Min–Max: 1.4–3.3)	(Min–Max: 1.8–22.8)	(Min–Max: 7.1–312.4)	(Min–Max: 0.3–15.8)	(Min–Max: 167.6–673.2)	(Min–Max: 2.4–12.9)
**Wild Rice**								
[18]	6	X = 238 ± 170	X = 3.3 ± 1.9	X = 7.8 ± 1.2	X = 561 ± 170	X = 5.5 ± 0.8	X = 120 ± 40	X = 24.7 ± 4.6
Our results	5	X = 294.6 ± 143.5	X = 12.6 ± 2.4	X = 17.9 ± 3.1	X = 1043.6 ± 155.1	X = 16.1 ± 2.3	X = 173.0 ± 11.1	X = 63.3 ± 17.2
		(Min–Max: 83.7–421.2)	(Min–Max: 10.5–16.6)	(Min–Max: 14.0–21.8)	(Min–Max: 873.7–1292.3)	(Min–Max: 13.9–19.8)	(Min–Max: 153.9–183.1)	(Min–Max: 44.6–85.4)
Rice								
[23]	9 *	X= 84.5	X = 1.1	X = 20.7	-	-	-	X = 23.4
[24]	56	-	X = 3.3 ± 0.9	X = 39.4 ± 17.6	-	X = 4.4 ± 1.4	X = 280 ± 200	X = 9.8 ± 2.8
			(min-Max:0.7–6.9)	(Min–Max: 11.8–90.6)	-	(Min–Max: 2.3–8.4)	(Min–Max: 0–630)	(Min–Max: 6.3–20.3)
[25]	23	X = 71.5 ± 7.3	X = 3.7 ± 0.2	-	-	X = 8.5 ± 0.5	X = 401 ± 92	X = 10.2 ± 0.3
[26]	30	-	X = 2.0 ± 0.5	-		X = 4.7 ± 0.6	X = 25.6 ± 10	X = 13.2 ± 2.1
Our results		X = 226.3 ± 160.6	X = 3.6 ± 2.8	X = 9.4 ± 7.0	X = 618.0 ± 498.4	X = 16.7 ± 10.0	X = 242.9 ± 140.4	X = 19.5 ± 15.0
(rice in total)		(Min–Max: 16.1–867.0)	(Min–Max: 1.3–16.6)	(Min–Max: 1.4–28.8)	(Min–Max: 7.1–1811.5)	(Min–Max: 0.3–53.0)	(Min–Max: 150.1–1174.1)	(Min–Max: 2.4–100.3)
**Rice Flour**								
[17]	4	-	X = 2.4	X = 31.2	-	X = 22.5	X = 117	X = 14.9
Our results	6	X = 192.1 ± 132.3	X = 2.4 ± 0.5	X = 8.7 ± 5.1	X = 571.4 ± 416.1	X = 15.7 ± 9.4	X = 381.2 ± 389.0	X = 15.2 ± 2.4
		(Min–Max: 65.7–426.3)	(Min–Max: 1.7–3.0)	(Min–Max: 4.5–18.1)	(Min–Max: 264.8–1385.1)	(Min–Max: 8.2–34.1)	(Min–Max: 188.7–1174.1)	(Min–Max: 12.4–19.0)

Min—minimum, Max—maximum, X—mean, *—every brand with five replications.

## Data Availability

The data presented in this study is available on request from the corresponding author.
